# Aflatoxin B1 Toxicity in Animal Models: Biomarker-Guided Mechanisms, Systemic Injury, and Precision Mitigation Strategies

**DOI:** 10.3390/toxins18080352

**Published:** 2026-08-17

**Authors:** Raza Mohai Ud Din, Xin Zhang, Salwa Eman, Ahmed A. Saleh, Mudathir Y. Abdulrahman, Hosameldeen Mohamed Husien, Shahab ur Rehman, Xiaodong Guo, Ning Chen, Mengzhi Wang

**Affiliations:** 1 Laboratory of Metabolic Manipulation of Herbivorous Animal Nutrition, College of Animal Science and Technology, Yangzhou University, Yangzhou 225009, China; mh24136@stu.yzu.edu.cn (R.M.U.D.); mh24126@stu.yzu.edu.cn (S.E.);; 2 College of Animal Science and Technology, Yangzhou University, Yangzhou 225009, China; 3 State Key Laboratory of Sheep Genetic Improvement and Healthy Production, Xinjiang Academy of Agricultural Reclamation Sciences, Shihezi 832000, China; 4 Animal and Fish Production Department, Faculty of Agriculture (Al-Shatby), Alexandria University, Alexandria City 11865, Egypt; 5 College of Veterinary Medicine, Albutana University, Rufaa 22217, Sudan; 6 Joint International Research Laboratory of Agriculture & Agri-Product Safety of MOE, Yangzhou University, Yangzhou 225009, China

**Keywords:** Aflatoxin B1, biomarker discovery, oxidative stress, gut–liver axis, animal toxicity

## Abstract

Aflatoxin B1 (AFB1) is a highly toxic mycotoxin which can be carried over into animal products and cause deterioration of livestock productivity when fed to livestock and wildlife. This review proposes a biomarker-guided framework for improving the early assessment of AFB1 exposure and toxicological responses in animal models. Oral exposure leads to the absorption of AFB1, which is bioactivated in the liver to the reactive AFB1-exo-8,9-epoxide that causes DNA and protein adduct formation, inflammation, mitochondrial apoptosis, and other effects. Cytochrome P450 activation and glutathione-dependent detoxification are in balance in determining susceptibility species, and this balance is different for poultry, pigs, ruminants, and rodents. In addition to traditional liver enzymes and histopathology, we highlight mechanistically informative biomarkers such as metabolites of aflatoxin, DNA and albumin adduct, lipid peroxidation products, antioxidant indices, cytokines, apoptotic markers, as well as signals involved in the Nrf2/NFκB pathway. AFB1 also damages the integrity of the intestinal barrier, the maintenance of the intestinal gut microbiota, reproductive function, growth performance, and development, thus creating a gut–liver-systemic toxic cascade. Finally, an assessment of stage-targeted interventions such as aluminosilicate binders, adsorbents derived from yeast, probiotics and nano-enabled interventions is conducted as viable tools for the reduction in exposure and injury. This review offers targeted mitigation strategies for early diagnosis of aflatoxicosis in animal production systems based on a biomarker approach.

## 1. Introduction

Aflatoxin contamination, primarily caused by Aspergillus spp, is a global food and feed-safety problem that poses public health and agricultural threats, particularly in tropical, subtropical and now also temperate regions [1,2]. Survey and meta-analytical data from decades of research show that levels of aflatoxins in processed products and in major staple crops, such as maize, groundnuts, sorghum and millet, have routinely exceeded regulatory limits for chronic exposure to humans and food-producing animals via dietary and contaminated feed chains, respectively [3].The threat is not static: the frequency and magnitude of climatic stressors such as high temperature, altered precipitation patterns, and droughts, which can make plants more susceptible to Aspergillus colonization and enable the production of mycotoxins, may increase [4], while the proportion of high-level contamination events occurs in areas which were previously classified as low-risk areas is likely to increase as a result of climate change [5]. Similar dynamics have been observed in the international assessments and sectoral analysis linking the future climate scenarios with the heightened aflatoxin risk to key food crops (particularly maize and groundnut) and the need to adapt to climate change in the value chain throughout the entire value chain [6,7]. Feed contamination affects animal production directly by inducing immunosuppression and making the animals more susceptible to infectious diseases by causing reproductive failure and by causing high morbidity/mortality in severe cases [5,8]. These biological effects can be monetized using the losses in production, increased veterinary costs, and the value of market rejection [9]. Animal-product (meat, milk, offal, eggs) surveillance confirms that carry-over and residues are present in real-world feeding conditions, and present both animal-health and downstream food-safety risks [10,11]. Aflatoxins have also been associated with impaired cell-mediated immune suppression and stunted growth in highly exposed populations after chronic exposure [12]. This also makes the problem One Health, as the contamination of the feed with AFB1 affects animal health, and can be passed on through food chains, creating a risk for human food safety and public health [13]. Aflatoxin is a target for regulatory harmonization, investment in rapid detection and biomarker development, cross-sectoral agriculture–veterinary–public health mitigation and is endemic in the environment, which is expected to become more of a problem under the increasing stresses of climate change. AFB1 has been widely reported to be the most toxic and mutagenic aflatoxin among the naturally occurring aflatoxins and the relative sequence of toxicity is AFB1 > AFG1 > AFB2 > AFG2 [14,15,16]. The hydroxylated form of AFB1 (AFM1) that can be excreted into milk is less toxic but is also a carcinogen and a public health concern as it can be exposed to humans through dairy products [17,18,19]. The International Agency on Research on Cancer (IARC) classifies AFB1 in the Group 1 category of human carcinogens, meaning there is high confidence that chronic exposure to AFB1 increases the risk of hepatocellular carcinoma [20]. Apart from cancer, chronic exposure to aflatoxins has been reported to adversely affect child development and immune function of exposed populations, further highlighting its importance as a food-safety and One Health issue [21]. There are interspecies differences in the susceptibility of livestock species to the toxicity of AFB1, which result from differences in hepatic enzymes that bioactivate AFB1, phase II enzymes that detoxify AFB1, rumen microbial metabolism, and antioxidant defenses [22]. Ducks are extremely sensitive to AFB1 as they readily convert it to the highly reactive epoxide form and have a relatively weak detoxification system, resulting in liver damage, immune system suppression, growth inhibition, and an increased incidence of deaths at higher exposures [23,24]. Swine are also susceptible, exposure has been associated with decreased feed efficiency and growth, impaired immune response, hepatobiliary metabolic changes and susceptibility to secondary infections [25,26]. Ruminants are, however, partially protected because some of the toxins are bio-transformed by ruminal microorganisms and some are bound by digesta but chronic exposure can reduce productivity and damage the liver. Importantly, AFB1 can be bio-transformed to AFM1 and released into milk, creating a serious potential food-safety risk [11,27]. Rodent models, particularly rats and mice, are the most commonly used experimental models for investigating AFB1 toxicity, because they have high and reproducible sensitivity to bioactivation of AFB1 and hepatocarcinogenesis, which is helpful for mechanistic research and chemoprevention studies [28,29,30]. AFB1 is the most important toxicological problem of aflatoxins, because it has the highest carcinogenicity, is able to undergo metabolic activation to form DNA-reactive metabolites, and there are interspecies differences in the metabolic activation and detoxification of AFB1 [31]. These mechanistic and species-specific variations are the key to proper risk assessment, biomarker development, and effective mitigation strategies in livestock production systems [32]. The significance of early warning of aflatoxin B1 (AFB1) exposure lies in the fact that the conventional approaches mainly verify the damage when the toxic harm has already been done, but biomarker science is in its infancy to improve early detection and mitigation assessment [32,33]. Veterinary practice assessment is typically done using feed analysis, clinical signs, serum liver enzymes (ALT and AST), and histopathology, although feed testing can be inaccurate because of aflatoxin distribution inequality in feed batches, and sampling error is a major cause of error [34]. ALT and AST are usually elevated once hepatic damage has already been done, and histopathology only confirms hepatocellular damage once tissues have been harvested, making these methods helpful in confirmation but not in early screening or screening at the herd level [35]. Recent advances in multi-omics have therefore shifted biomarker discovery toward earlier and more mechanistic signals: metabolomics has revealed perturbations in amino acid metabolism, lipid oxidation, and bile-acid-related pathways; transcriptomic and proteomic studies have identified xenobiotic-response, detoxification, mitochondrial, and liver-development changes, and miRNA profiling is increasingly being explored as a complementary early indicator of AFB1 toxicity [36,37]. Another promising frontier is the microbiome: AFB1 can alter gut microbial composition and intestinal barrier integrity, and fecal microbiota profiling has already been proposed as a non-invasive approach for screening exposure and resilience in exposed animals [38,39]. A paradigm shift in conventional diagnostics to biomarker-based diagnosing is a shift towards precision livestock health management. By diagnosing at an early stage, feed can be detoxified, nutritional supplements provided, and the microbiome regulated before irreversible tissue damage occurs. The present review helps to create a mechanism-based, species-specific model of aflatoxin B1 (AFB1) toxicity by integrating biomarkers, molecular pathways, and systemic effects between the species. It extends beyond classical definitions of contamination or hepatotoxicity and combines exposure biomarkers, oxidative stress, inflammation, and apoptotic signaling into a unified toxicological cascade. It is important to note that new interventions, including adsorbents, probiotics, nano-enabled systems, and multimodal strategies, are superimposed to discrete phases of this cascade, which provides a translational and stage-selective solution to reduce aflatoxicosis in animal production systems.

### Literature Search Strategy and Study Selection

This article was conducted as a narrative review with a structured literature search to comprehensively summarize current evidence on aflatoxin B1 (AFB1)-induced toxicity, biomarker discovery, systemic toxicological effects, and mitigation strategies in animal models. Although a formal systematic review protocol was not applied, the literature search and study selection were performed using predefined criteria to improve transparency and reproducibility, consistent with recommendations for high-quality narrative reviews [40].

A comprehensive search was conducted in PubMed, Web of Science Core Collection, Scopus, and Google Scholar for studies published from database inception to March 2026. Additional relevant publications were identified through manual screening of reference lists from key review articles and highly cited original studies. The search strategy combined medical subject headings (MeSHs) and free-text terms using Boolean operators, including “*Aflatoxin B1*” OR “*AFB1*”, “*animal models*”, “*livestock*”, “*poultry*”, “*ruminants*”, “*swine*”, “*rodents*”, “*biomarkers*”, “*DNA adducts*”, “*albumin adducts*”, “*oxidative stress*”, “*inflammation*”, “*hepatotoxicity*”, “*gut–liver axis*”, “*mitigation*”, “*adsorbents*”, “*probiotics*”, and “*mycotoxin detoxification*” [41].

Peer-reviewed original research articles, controlled animal experiments, mechanistic studies, comparative toxicology investigations, and authoritative review articles published in English were considered eligible. Studies were included if they investigated AFB1 exposure in livestock or laboratory animal models and reported toxicokinetic characteristics, molecular mechanisms, biomarkers of exposure or toxicity, pathological outcomes, or mitigation strategies. Studies focusing exclusively on other mycotoxins, lacking sufficient methodological information, conference abstracts, editorials, duplicate publications, or non-peer-reviewed reports were excluded.

Titles and abstracts were initially screened for relevance, followed by full-text assessment of potentially eligible articles. Data extracted from the selected studies included animal species, experimental model, AFB1 dose and exposure duration, biological matrix, analytical methods, biomarkers evaluated, mechanistic pathways, toxicological outcomes, and mitigation approaches. The studies summarized in tables were selected based on their scientific quality, methodological rigor, relevance to the review objectives, and their contribution to comparative interpretation across animal species. Owing to the substantial heterogeneity of experimental designs, animal species, exposure conditions, and outcome measures, findings were synthesized qualitatively without formal meta-analysis or risk-of-bias assessment.

## 2. Chemical Structure and Physicochemical Properties of Aflatoxin B1 (AFB1)

*Aspergillus flavus* and *Aspergillus parasiticus* primarily produce aflatoxin B1 (AFB1), a very toxic secondary metabolite, which is a major contaminant of crops and feed in warm and damp climates [42]. Its molecular formula is C_17_H_12_O_6_ and its molecular weight is approximately 312.27 g/mol, reflecting a relatively small but highly reactive molecule [43,44]. AFB1 is a difuranocoumarin, structurally a coumarin (benzopyrone) nucleus containing a bifuran moiety, a planar and highly conjugated aromatic system [45]. The fused-ring structure is known to stabilize the compound physicochemically and to promote the interaction with biological macromolecules, including DNA and proteins [46]. The terminal furan ring (and the C8C9 double bond in particular) is the major toxicological determinant and the major site of bioactivation by the cytochrome P450 [47]. These structural features cause AFB1 to be regarded as the strongest mutagenic and carcinogenic aflatoxin and a Group 1 human carcinogen [46]. Its high fluorescence is also linked to the intact lactone-containing structure, commonly utilized in the analysis of the structure, and typically seen at excitation wavelengths of 365 nm [48]. From a physicochemical standpoint, AFB1 is sparingly soluble in water but readily soluble in polar organic solvents such as methanol, chloroform, and acetone [49]. Its logP is about 1.60, which is moderately lipophilic, and thus it will diffuse passively across the membrane and be absorbed by the gastrointestinal tract easily [50]. AFB1 is also bound by plasma proteins, e.g., albumin, and this influences its systemic distribution and retention in the circulation [51]. The molecule is quite stable over a broad pH range; but in highly alkaline environments, lactone-ring hydrolysis/opening is induced to produce less toxic derivatives [52]. Photochemical degradation of AFB1 is possible under UV light, but the rate of degradation is extremely dependent on irradiation intensity, wavelength, matrix structure, and exposure time, so UV degradation is generally a controlled-laboratory or surface-treatment technology, rather than a large-scale agricultural detoxification technology [53]. Non-ruminant systems have limited enzymatic detoxification of AFB1, but the rumen partially bio-transforms the toxin, though not completely and inconsistently [54]. AFB1 belongs to the B-series of aflatoxins, which is blue in the UV light, and G-series of aflatoxins, which is green in the UV light, because of the differences in chemical structure and oxygenation pattern [55]. Among the naturally occurring aflatoxins, AFB1 is the most toxic since its structure is ideally positioned to be metabolically activated, bind DNA and persist, all of which help to make it highly mutagenic and carcinogenic [56]. Major change endpoints can alter toxic potency: AFM1 hydroxylation can alter its activity and allow milk carry-over, and saturation of the double bond in AFB2 is more likely to decrease carcinogenic potential, which is a clear structure–activity relationship in aflatoxin toxicology [31]. In addition to milk, AFM1 and aflatoxin residues in eggs represent a potential food safety concern and should be considered when evaluating carry-over in animal-derived foods [57].

## 3. AFB1 Toxicokinetic and Bioactivation in Animals

The major route of exposure of AFB1 in animals is by consuming contaminated feed, and oral exposure is the most common route of exposure in poultry, pigs, and dairy cattle [26,58,59]. AFB1 is easily absorbed across the intestinal mucosa of monogastric animals largely due to its small size and it being lipophilic, and thus is readily absorbed across the intestinal mucosa of monogastric species such as pigeon, rat, sheep, and goat [23,60]. Following intestinal absorption, AFB1 enters the portal circulation and is delivered to the liver, where first-pass biotransformation becomes the critical determinant of systemic toxicity in animals [15]. Part of the absorbed toxin may also be degraded in the gut before reaching the liver since rumen microorganisms are capable of partially degrading AFB1, although this detoxification is incomplete and differs by species and rumen conditions [61]. In pigs, serum AFB1-lysine adducts are already known as an internal-dose biomarker, and adsorption-based interventions have been shown to lower adduct formation, which confirms their usefulness in monitoring exposure in livestock [62]. Both serum AFB1-albumin adducts and blood AFB1-DNA adducts are being used to assess chronic exposure, further supporting albumin-related adducts as biologically significant exposure-recording events in ruminants [63].

After absorption, AFB1 is subjected to extensive hepatic biotransformation, with the liver being the primary site of phase I activation and phase II detoxification of AFB1 in livestock species [64]. Recent mechanistic studies in cattle indicate that cytochrome P450 systems, in particular CYP1A1 and CYP3A74, are central in AFM1 metabolism and in the regulation of AFM1 formation, AFBO synthesis and cytotoxicity [15]. The products of phase I metabolism include a variety of hydroxylated and related products, such as aflatoxicol, AFM1, AFQ1, and hydrated products including AFB2a, O-demethylated products such as AFP1, reduced products such as aflatoxicol, and the highly reactive epoxide intermediates, AFB1-endo-8,9-epoxide and AFB1-exo-8,9-epoxide [59,65]. The exo-8,9-epoxide is the most important bioactivated metabolite of AFB1 that is responsible for genotoxicity and carcinogenicity. This is the metabolite that is produced via the epoxidation catalyzed by CYP450 and is the major electrophilic intermediate between hepatic metabolism and downstream DNA damage in animals, including bovine hepatocyte models and comparative hepatocyte studies across species [15,66]. The exo-epoxide can easily attack the nucleophilic sites in DNA, proteins, phospholipids, and other cellular macromolecules, thus causing cellular dysfunction, membrane damage, and toxic harm [67]. The alkylation of DNA takes place mostly at the N7 position of guanine, and it results in the formation of the pro-mutagenic AFB1-N7-Gua adduct. This lesion is also a canonical marker of AFB1 exposure, and it has been shown by mechanistic studies of the aflatoxin–DNA reaction chemistry [68]. Since the N7-guanine adduct is unstable, it can be subjected to further processing to form more persistent ring-opened products, particularly AFB1-formamidopyridine (AFB1-FAPY) adducts. The lesions are more enduring in DNA and noteworthy because animal studies show that the lesions are highly mutagenic in vivo [69]. The one major mutational impact of this adduct chemistry is the G-to-T transversion at the third nucleotide of codon 249 of the p53 tumor suppressor gene, which is the paradigm molecular signature of AFB1-related hepatocarcinogenesis. Despite this hotspot having been defined in both studies of human hepatocytes and tumors, it is the best-established downstream readout of the same bioactivation-to-adduct pathway that is also observed in animal mechanistic systems [70]. Detoxification is competing with AFB1 bioactivation, as species selectivity is extremely dependent upon the extent of disparity on the expression and catalytic functionality of the biotransformation enzymes. Recent comparative reviews point out the fact that AFB1 toxicity is not species fixed, but rather it does reflect the efficiency with which an animal partitions the toxin between activation and detoxification routes [22]. The major route of protection is conjugation of AFB1-exo-8,9-epoxide (AFBO) with glutathione (GSH), which is catalyzed by glutathione S-transferases (GSTs), followed by additional processing through the mercapturic acid pathway to more water-soluble excretion products [71]. If this detoxification pathway is compromised, species are at risk because the AFBO may persist long enough to damage cellular macromolecules, causing genotoxicity. This is more so in sensitive animal models where the GST functionalities are compromised or less effective against the reactive epoxide [72,73]. Similar to GSH conjugation, AFBO may also be subjected to epoxide hydrolase-mediated conversion to AFB1-dihydrodiol, which can, in turn, break down to AFB1-dialdehyde [74]. This dialdehyde is biologically significant in that it is a binding molecule of proteins, especially lysine residues in albumins, and thus causes acute toxicity and protein adduct formation. Protein adducts are currently considered as useful internal-dose markers in animal investigations [63]. The balance between activation and detoxification is therefore strongly associated with species resistance. Animals with higher GST capacity or higher rate of clearance of detoxified products are more resistant. This pattern explains why rodents, poultry, pigs, and ruminants differ so markedly in AFB1 susceptibility and biomarker profiles. In pigs, porcine CYP2A19 has recently been shown to play a key role in hepatic AFB1 bioactivation [75]; in poultry, turkey GST studies show deficient AFBO detoxification [73]; in ducks, unusually high hepatic AFB1-dihydrodiol production helps explain their extreme sensitivity [74]; and in mice, rapid detoxification is associated with strain resistance. In general, the phase of absorption and hepatic activation should be considered as the biological bottleneck that defines downstream damage. A combination of passive intestinal uptake, the formation of AFBO under the influence of CYP450, and competing GST-dependent detoxification explain why a combination of early biomarkers of exposure and metabolic stress is especially informative in animal model studies.

Species-specific susceptibility to AFB1 is largely determined by differences in its biotransformation rather than by the toxicant itself. Although the principal metabolic pathways are conserved among vertebrates, substantial interspecies variation exists in the expression and catalytic activity of cytochrome P450 enzymes responsible for AFB1 bioactivation, as well as glutathione S-transferase-mediated detoxification pathways. Consequently, the balance between metabolic activation to the highly reactive AFB1-exo-8,9-epoxide and detoxification differs markedly among animal species, leading to pronounced differences in hepatotoxicity, carcinogenicity, and biomarker responses. Therefore, findings obtained in one species should not be directly extrapolated to another without considering these physiological and metabolic differences [22].

**Figure 1:**
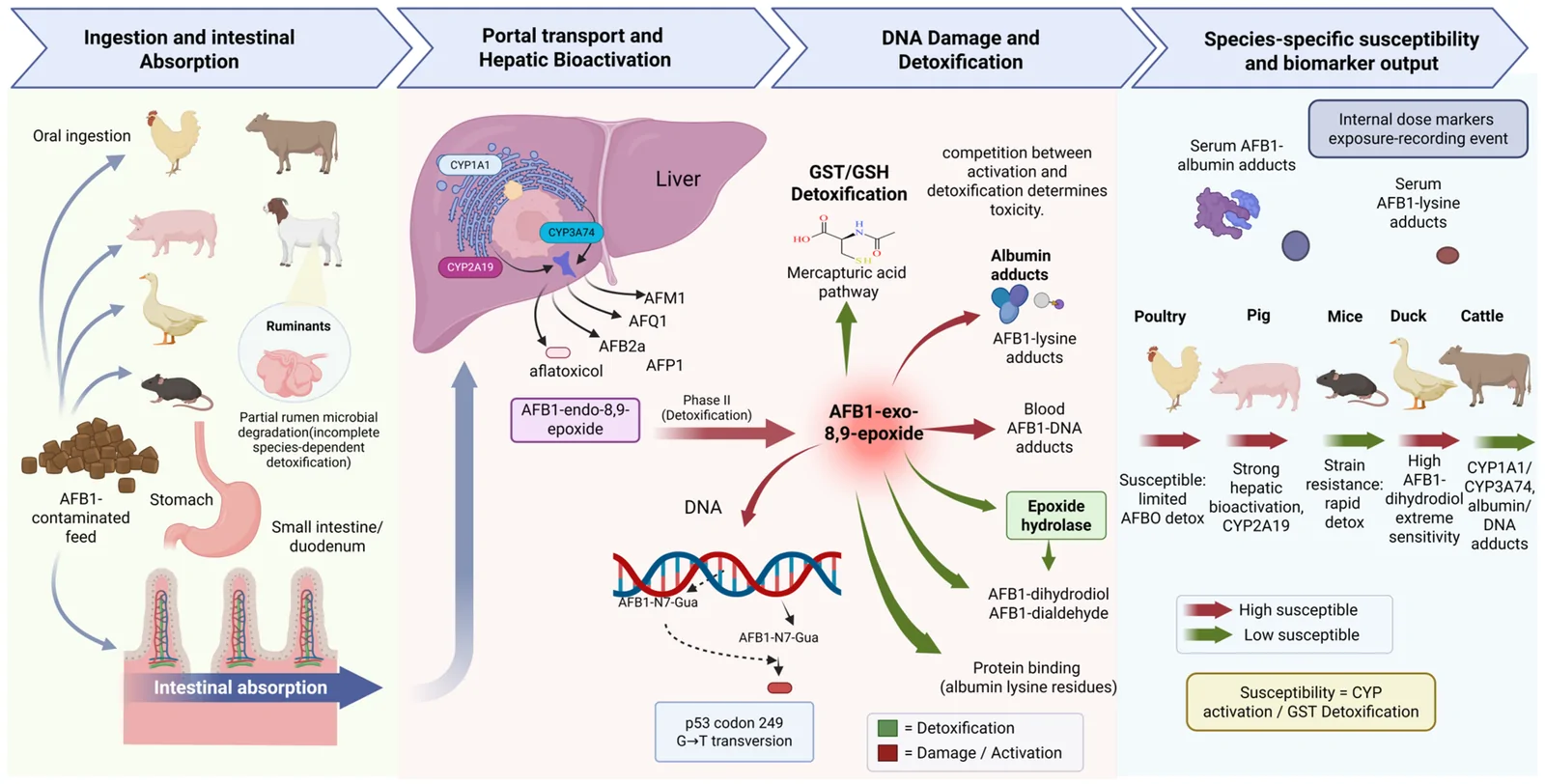
Mechanistic overview of aflatoxin B1 (AFB1) absorption, hepatic bioactivation, and downstream toxicity in animal species. Following oral ingestion of contaminated feed, AFB1 is absorbed in the small intestine and transported via the portal circulation to the liver, where cytochrome P450 enzymes (e.g., CYP1A1, CYP3A74, CYP2A19) convert it to the reactive AFB1-exo-8,9-epoxide. This metabolite forms DNA (AFB1-N7-Gua, AFB1-FAPY) and protein (albumin, lysine) adducts and contributes to mutational events such as p53 codon 249 transversion. Detoxification occurs via glutathione S-transferase (GST)-mediated conjugation and epoxide hydrolase pathways. The balance between bioactivation and detoxification determines species-specific susceptibility (poultry, pigs, cattle, ducks, mice) and is reflected by circulating biomarkers including AFB1-albumin, AFB1-lysine, and DNA adducts, as well as metabolites such as AFM1 and AFQ1.

The graphical representation of absorption, hepatic activation, and formation of AFB1-epoxide is shown in Figure 1.

## 4. Biomarkers of AFB1 Exposure and Early Injury

It should be noted that not all biomarkers discussed in this review are uniquely specific to AFB1 exposure. Several biochemical, oxidative stress, inflammatory, and physiological biomarkers (e.g., ALT, AST, MDA, SOD, CAT, GPx, and pro-inflammatory cytokines) are general indicators of tissue injury and may also be altered by other toxicological, nutritional, or environmental stressors. However, the studies included in this review were conducted under controlled experimental conditions, in which AFB1 exposure was the primary experimental variable, thereby minimizing potential confounding factors. Consequently, these biomarkers are discussed as AFB1-associated response biomarkers that provide insight into the biological effects and mechanistic pathways of AFB1 toxicity in animal models, rather than as biomarkers uniquely diagnostic of AFB1 exposure. Exposure-specific biomarkers, such as AFB1–albumin adducts, AFB1–N7-guanine DNA adducts, and AFM1, remain the most specific indicators of AFB1 exposure.

### 4.1. Exposure Biomarkers: AFB1 Metabolites, DNA Adducts, Albumin Adducts

In animal aflatoxin research, biomarkers are usually more informative than feed contamination data alone because AFB1 biotransformation and susceptibility differ markedly among species [22,26]. The most useful exposure biomarkers are excreted metabolites for recent intake, DNA adducts for biologically effective dose, and albumin/lysine adducts for longer-term integrated exposure [76]. In broiler chickens, AFB1 was identified as an optimal biomarker in plasma and excreta in a detoxifier-efficacy study, and a separate chicken biomarker method found AFB1-N7-Gua only in excreta [77]. AFM1 was detected in both ileal content and excreta and was about five times higher in excreta, indicating that excretion is a major elimination route in this model [78]. For pigs, urinary AFB1 and AFM1 are established short-term exposure biomarkers. A controlled feeding study reported that about 30% of the oral dose was recovered in urine as AFB1 plus AFM1, with an AFB1-to-AFM1 ratio of about 23%, and later pig biomarker reviews reported urinary recoveries of 20–48% depending on dose and sampling conditions [79,80].

AFB1-lysine adducts are formed from albumin-bound aflatoxin adducts after enzymatic digestion, so they represent a longer-integrated exposure marker than free urinary metabolites [32]. In pigs, AFB1-lysine has been validated in serum and plasma by LC-MS/MS as a biomarker of AFB1 exposure [81]. In dairy cattle, aflatoxin-albumin adducts in blood have been proposed as a marker of chronic aflatoxin exposure and were compared with AFM1 in milk [82]. A cattle study in 2025 further expanded the biomarker approach by evaluating serum AFB1-albumin adducts together with blood AFB1-DNA adducts for assessment of individual chronic exposure [63]. Because ruminal degradation of AFB1 varies with species, feed type, and ruminal conditions, internal-dose biomarkers are still needed in cattle rather than relying on feed contamination alone [54,61].

DNA adducts are the most mechanistically informative exposure biomarkers because AFB1 is bioactivated to the reactive exo-8,9-epoxide, which preferentially forms N7-guanine adducts in DNA [83,84]. In rats, urinary AFB1-N7-Gua has been validated as a biomarker of internal aflatoxin dose and liver DNA adduct formation [83,85]. The value of measuring DNA adducts is that it links aflatoxin exposure to the earliest mutagenic event rather than to a later clinical outcome [86]. Among aflatoxin DNA lesions, AFB1-N7-Gua is the major adduct in rodent and human systems, and it is chemically unstable, rapidly released from DNA, and excreted in urine [87]. The ring-opened AFB1-FAPY adduct persists much longer than AFB1-N7-Gua, so it better reflects longer-lasting genomic injury [88]. Albumin adducts complement DNA adducts because they remain in circulation longer and provide a wider exposure window than rapidly excreted urinary metabolites [32,76]. Serum AFB1-albumin adducts have been described as the most reliable biomarker for chronic aflatoxin exposure in comparative work, and in cattle and pigs they provide a biologically meaningful bridge between absorbed dose, hepatic bioactivation, and systemic exposure burden [89]. Taken together, a combined biomarker strategy provides the strongest framework for linking contaminated feed, species-specific metabolism, and early injury in animal models. Animal matrices used for monitoring AFB1 exposure biomarkers and their practical interpretation are shown in Table 1.

### 4.2. Early Injury Biomarkers: ALT, AST, ALP, MDA, GSH, SOD, CAT

In animal AFB1 studies, early injury biomarkers are useful because AFB1 hepatotoxicity is driven by mitochondrial dysfunction, oxidative stress, and loss of antioxidant defenses, so biochemical changes can appear before overt structural damage becomes obvious [92]. Across poultry, duck, rat, and other experimental models, the most reproducible toxic response is a simultaneous increase in serum liver enzymes, depletion of antioxidant defenses, and accumulation of lipid peroxidation products [93]. A pattern of these biomarkers suggests that AFB1 injury is not mass-scale structural damage but rather the impairment of hepatocyte membrane integrity, mitochondrial redox homeostasis, and intracellular detoxification systems [94]. The most popular first-line markers of hepatocellular injury are ALT and AST [95]. ALT is predominantly a cytosolic enzyme; AST is found in the cytosol and mitochondria, such that an increase in AST may often be indicative of deeper injury and mitochondrial involvement [96]. Alternation of the ALT and AST by exposure of ducklings to AFB1 was accompanied by liver injury and derangement of the bile metabolism, which demonstrate that transaminase elevation is initial hepatocellular dysfunction in an avian model [97,98]. Rodents remain valuable experimental models for investigating AFB1 toxicity; however, important metabolic differences exist between rodents, livestock species, and humans. Consequently, rodent findings should be interpreted within the context of species-specific AFB1 biotransformation pathways. Rats acutely exposed to AFB1 also had their ALT and AST impacted, which argues in favor of the enzymes as being sensitive biomarkers of early hepatic toxicity across animal species [99]. AST is among the chief serum enzymes that are elevated in aflatoxicosis, and it is utilized to indicate the presence of hepatic injury in ruminants [100]. Studies conducted in rabbits observe an increase in ALT following exposure to AFB1, and the increase is usually accompanied by oxidative stress and degraded protein status [101,102]. In sheep, exposure to AFB1 at low doses is likely to induce minimal or no change in ALT in concert with AST, but will do so at low doses in goats [103,104]. Mechanistically, bioactivation of AFB1 induces the formation of reactive intermediates, which damage mitochondria and destabilize hepatocyte membranes, leading to leakage of ALT and AST into serum [92]. Once membrane permeability increases, these enzymes escape into serum, which makes them practical readouts of both cell injury and the intensity of intracellular stress [105]. This is particularly critical when using animal models because the progression of enzyme changes in most cases precedes the development of severe necrosis; it is possible to detect subclinical toxicity in poultry and livestock feeding trials [106]. Another layer to the injury profile of that it is compared to ALT and AST since it is more strongly correlated with hepatobiliary and bile-canalicular dysfunction than with mere leakage of the hepatocytes [107]. Mechanistically, ALP comes in handy as it tends to increase when AFB1 damage is not a result of membrane leakage, but rather due to biliary stress, impaired canalicular transport, and altered hepatocyte polarization [108]. So, in animal models, a concomitant elevation of the levels of ALT, AST, and ALP is an indicator of a broader hepatic syndrome, which is a cytosolic injury, perturbation of mitochondria, and altered bile management rather than a single, well-defined lesion [109].

MDA is the most commonly reported biochemical readout of lipid peroxidation, and one of the simplest signatures of AFB1-induced oxidative damage, as AFB1 activates the production of free radicals that attack lipids in membranes [110]. MDA can be considered a reflection of phospholipid damage by ROS formed after AFB1 bioactivation. Increasing MDA levels indicates that cellular and organelle membranes are already exposed to oxidative stress [111]. Since MDA is a downstream product of lipid oxidation, it reflects cumulative oxidative burden, but not a transient stress signal, and a high level of MDA tends to indicate cumulative oxidative burden, but not a momentary stress signal [112]. AFB1 exposure of animal models demonstrates that MDA is an early injury biomarker. Studies have shown that the exposure of broilers to AFB1 causes an increase in the amount of MDA, which is an indication of lipid peroxidation and oxidative damage to the membranes [113]. AFB1 has been demonstrated to cause oxidative damage markers like MDA in swine, showing the toxin promotes oxidative damage in swine membrane lipids [114]. Research studies of dairy cows have shown that exposure to AFB1 leads to an increase in MDA and a decrease in SOD-related antioxidant balance, indicating systemic oxidative stress in ruminants [115]. Hepatic and cardiac rat studies show that hepatic and cardiac MDA increase following acute exposure to AFB1 [99]. Studies with rabbits have reported a significant increase in ROS, MDA, and 4-HNE after exposure to AFB1, with a pronounced lipid peroxidation in this species [116]. GSH is at the center of aflatoxin defense, having a role in maintaining intracellular redox balance and in providing the thiol donor necessary to facilitate GST-mediated conjugation of the reactive AFB1-8,9-epoxide [117]. Mechanistically, the GSH reduction results in a reduced ability of the liver to neutralize the reactive epoxide, leading to an increase in propensity of the DNA and protein adducts to form and an increase in risk of downstream genotoxic injury occurring [118]. A loss of GSH is a frequent theme in the larger animal literature, which is a loss of detoxification capacity in the cell [119]. The tests on cattle have shown that GSH depletion occurs with aflatoxicosis, which is not surprising considering that ruminants still experience the loss of GSH, despite the partial detoxification of the rumen [100]. SOD and CAT are some of the most commonly used enzymatic antioxidant markers as they will indicate whether or not the animal still has enough first-line capacity to counter reactive oxygen species [120]. SOD transforms the superoxide to hydrogen peroxide, whereas catalase (CAT) catalyzes the decomposition of hydrogen peroxide (H_2_O_2_) into water and molecular oxygen, thereby protecting cells from oxidative damage [121]. Mechanistically, a fall in SOD implies that the removal of superoxide cannot be as efficient, whereas a fall in CAT allows the persistence of hydrogen peroxide and its ability to feed other reactions leading to the production of radicals. It is this disruption of the enzymatic defense that allows the self-amplifying oxidative cascade that increases membrane damage, mitochondrial dysfunction, and apoptosis following AFB1 exposure [122].

These markers, together with the others, make up a coherent panel of early injury in the animal models. The ALT, AST, and ALP are best interpreted as the hepatocellular/hepatobiliary injury-related markers, but the MDA, GSH, SOD, and CAT describe oxidative stress and antioxidant failure. An informative interpretation of the combination of the biomarkers should be their pattern, rather than a single number [67]. Early injury biomarkers of AFB1 toxicity in animal studies are given in Table 2.

### 4.3. Mechanism-Linked Biomarkers: Inflammatory Cytokines, Apoptosis Markers, Nrf2/NF-κB-Related Markers

Mechanism-linked biomarkers are among the most informative early-response indicators in AFB1-exposed animals because they appear downstream of exposure and reflect the actual biological pathways that drive tissue injury [126]. AFB1 typically induces a pro-inflammatory response with an increase in IL-1b, IL-6, and TNF-α, and in several poultry datasets also with increasing IL-18 and IFN-γ; thus, cytokine activation is a common early response to exposure [127]. In the case of research in bovine liver cells, it has been found that AFB1 activates an inflammatory transcriptional program dominated by TNF signaling via NF-κB and interferon-gamma response, which is species-specific in the case of bovine and ruminant hepatocytes [64]. Inflammatory liver injury in sheep studies has been reported to be linked with increases in TNF-alpha and IL-6 in response to AFB1-linked increases in the plasma concentration of AFB1 [104]. Exposure of AFB1 to rabbit intestine/kidney/liver models increases TNF-α, IL-1β, IL-6, and IFN-γ, which proves the existence of evident inflammatory injury and immune response activation [128]. The corresponding changes have been noted in the thymus and bursa of Fabricius of broiler chickens, where AFB1 regulates the expression of Bax, Bcl-2, and caspase-3, and it is indicated that lymphoid organs are also highly sensitive to apoptotic injury [129]. Reduction in Bcl-2 and increased Bax and caspase-related signaling have also been suggested to mediate liver injury induced by AFB1 in ducks, again supporting a mitochondria-centered apoptotic pathway [130]. In intestine models, AFB1 increases Bax and caspase-3 and decreases Bcl-2, which demonstrates AFB1 does not cause apoptosis in the liver but, rather, is also a contributor to barrier injury [131]. A significant part of the AFB1 toxicology is the apoptosis markers, since the toxin leads to the destruction of mitochondria and caspase-dependent cell death in the liver, intestine, kidney, thymus, and several other target tissues. The most frequently used apoptosis markers in the animal AFB1 studies include Bax, Bcl-2, Bax/Bcl-2 ratio, cytochrome c, caspase-9, caspase-3, and p53, by virtue of the fact that they are all useful in describing the mitochondrial death pathway [132]. Exposure of mice to AFB1 has been shown to increase caspase-3 activity and the extent of apoptosis in genetically susceptible strains, which suggests that genetic background can determine the magnitude of the apoptotic response [133].

Nrf2 and NF-κB can be framed as two tightly linked but functionally opposite regulatory systems, with Nrf2 governing antioxidant defense and NF-κB driving inflammatory transcription [134]. In chicken liver models, AFB1 has been shown to suppress Nrf2 signaling while increasing NF-κB p65 activation, whereas protective phytochemicals such as astaxanthin, phillygenin, curcumin, and α-lipoic acid restore Nrf2 activity and dampen NF-κB signaling [135]. Duck studies show the same overall pattern: resveratrol and curcumin alleviate AFB1-induced liver or ileum injury by restoring Nrf2-related defense and suppressing NF-κB-linked inflammatory signaling, while reducing oxidative stress and tissue damage [136]. The causal role of Nrf2 has been demonstrated in rodents: Nrf2-knockout rats are much more vulnerable to AFB1 toxicity, show greater hepatotoxicity, and accumulate more AFB1-N7-guanine DNA adducts than wild-type rats after exposure [30]. Mechanism-linked biomarkers of AFB1 toxicity in animal studies are given in Table 3. Although numerous biomarkers have demonstrated promising diagnostic potential in experimental animal models, relatively few have undergone comprehensive validation for routine veterinary or field applications. Biomarker performance may vary according to animal species, biological matrix, exposure duration, analytical platform, and environmental conditions. Consequently, many currently reported biomarkers should be regarded as exploratory rather than fully validated diagnostic tools. Future studies should establish standardized analytical protocols, diagnostic thresholds, sensitivity, specificity, and inter-laboratory reproducibility to facilitate translation into practical livestock health monitoring.

## 5. Systemic Effects

### 5.1. Hepatic Toxicity: The Central Organ of Injury

The liver is the main target organ for aflatoxin B1 in animals because absorbed toxin reaches it through the portal circulation, and it is also the principal site where cytochrome P450 enzymes carry out AFB1 bioactivation [137]. The entry of AFB1 into hepatocytes is metabolized to the reactive AFB1-8,9-epoxide (AFB1-exo-8,9 epoxide) which can bind DNA and other cellular macromolecules and thereby triggering the downstream cascade of oxidative stress, inflammation, and cell death that characterize aflatoxicosis [92,138]. At the tissue level, aflatoxin injury generally switches between reversible hepatocellular stress and overt structural damage, which initially involves hepatocyte swelling, fatty degeneration, and disruption of hepatic cord organization, followed by necrosis, inflammatory infiltration, and hyperplasia of the bile ducts [139,140]. Even a single acute exposure can cause permanent liver damage that may be identified days after exposure cessation in rats. The injury does not need to be resolved promptly after termination of exposure in rats and with high levels of lipid peroxidation and apoptosis [99]. A fibrotic remodeling is now recognized to be a part of the chronic hepatic response to AFB1 in animals, and recent work with ducklings has described hepatic steatosis and fibrosis involving PPARa- and TGFb-related pathways [141]. One of the earliest biochemical consequences of AFB1 exposure in animal liver is suppression of protein synthesis, so impaired synthetic function appears very early in aflatoxicosis [142]. Rat liver studies showed that AFB1 reduces hepatic expression of albumin and other acute-phase-related proteins, indicating that the toxin alters normal protein production rather than causing only later structural injury [143]. Bile metabolism is another major downstream target of AFB1 in animals, particularly in duck and rodent models [97]. The duck studies reported increased ALT, AST, and ALP together with altered expression of bile-acid-related genes such as CYP7A1, CYP8B1, and BSEP, which supports a cholestatic injury pattern combined with oxidative stress [93]. In mice, AFB1 induced hepatic bile duct proliferation, oxidative stress, inflammation, and liver injury, while also causing gut microbiota dysbiosis, reduced fecal bile salt hydrolase activity, increased hepatic bile acid synthesis, and suppression of intestinal FXR/FGF-15 signaling [144]. Chronic AFB1-related liver injury can progress from inflammation and oxidative stress into preneoplastic and neoplastic change, and low-dose chronic exposure in Sprague–Dawley rats has been reported to produce toxicant-associated fatty liver disease with fatty infiltration, multiple foci, preneoplastic lesions, and adenomas [145]. At the molecular level, AFB1 is bioactivated to the exo-8,9-epoxide, which forms unstable N7-guanine adducts and more persistent FAPY lesions; these long-lived DNA lesions promote mutation fixation and genomic instability [146]. The best-known mutational signature in AFB1-associated hepatocarcinogenesis is the p53 codon 249 G-to-T hotspot, and mechanistic studies show that AFB1-related lipid peroxidation can inhibit DNA repair and increase mutational sensitivity, linking oxidative injury to hotspot fixation [147]. Mouse studies further show that deficient repair of AFB1-induced Fapy lesions increases mutation frequency and HCC susceptibility, which supports the idea that impaired repair is a key step in AFB1-driven carcinogenesis [148].

### 5.2. Gastrointestinal and Barrier Dysfunction

The first anatomical and functional barrier met by dietary AFB1 is the intestine, and current animal studies do support the concept that the intestine is a direct target organ of dietary AFB1, and not just a passive route of absorption [149,150]. In animal models, AFB1 may damage the small intestine, both through direct toxicity to the epithelial cells and by local bioactivation, due to the ability of intestinal tissues to contribute to the metabolic activation of AFB1 and subsequent cellular injury [151]. Intestinal mucosal injury is a common lesion in animal models, which is typically observed as a loss of the villus-height-to-crypt-depth ratio, disorganization of the epithelial layer, and shedding or apoptosis of the absorptive cells [152]. Exposure to AFB1 in broiler research has been repeatedly associated with the deterioration of intestinal surface morphology and the reduced ratio of the villus-height-to-crypt-depth, which indicates the loss of absorptive surface area and digestive efficiency [153]. The main tight-junction proteins, which have been perturbed in animal studies, include those of ZO-1, occludin, and claudins, which are essential in animal studies in the context of paracellular sealing and epithelial polarity [154]. AFB1 suppressed FXR in mice and ZO-1, occludin, and claudin-1, linking failure of the barrier with disturbed bile-acid signaling [155]. Pigs fed a chronic low level of AFB1 inhibited the expression of ZO-1 and increased the serum levels of the intestinal barrier, which is the typical damage and leakage of the intestinal barrier [25]. Functionally, AFB1-related structural damage can translate into higher intestinal permeability, but the size of that effect varies with species, dose, exposure duration, and the assay used to measure leakiness [156].

AFB1 exposure alters the composition and function of the intestinal microbiota, contributing to intestinal dysbiosis and impaired gut health [38]. In rats with AFB1 disruption the profile of gut microbial populations was disrupted, and was linked with overgrowth of *Alloprevotella* which, as the authors established, is associated with gut injury and carcinogenic potential [157]. The exposure to AFB1 in pigs has been associated with an increase in abundance of *Escherichia coli* in the colon, a decrease in intestinal antioxidant capacity and an increase in the expression of pro-inflammatory cytokines [65,114].

**Figure 2:**
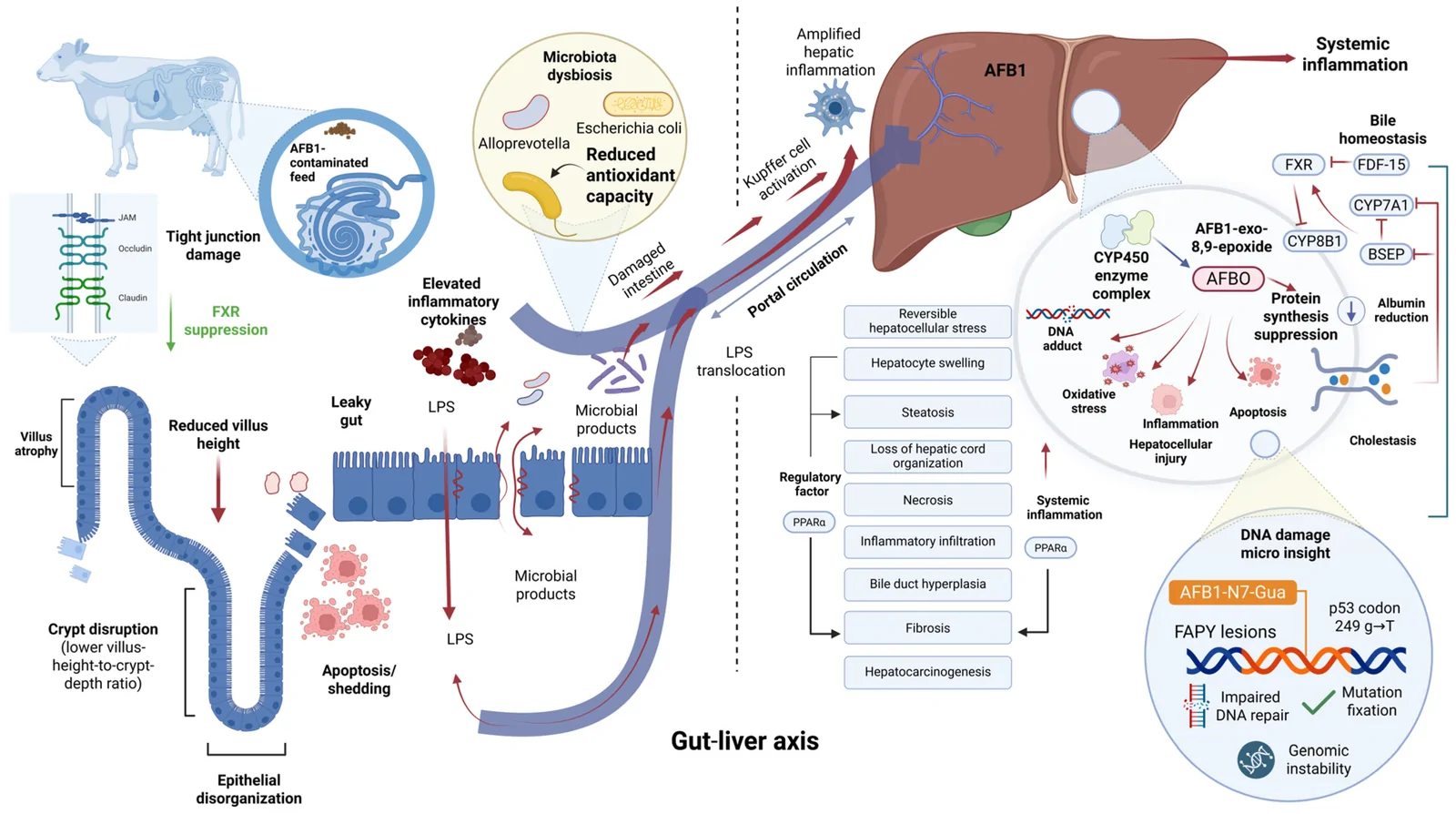
Mechanistic overview of AFB1-induced systemic toxicity in animals. Dietary AFB1 is absorbed in the intestine and delivered to the liver, where CYP450-mediated bioactivation to AFB1-exo-8,9-epoxide drives oxidative stress, DNA adduct formation, impaired protein synthesis, cholestasis, and progressive hepatocellular injury leading to fibrosis and carcinogenic change. In parallel, AFB1 disrupts intestinal integrity by damaging villi and tight junction proteins, inducing microbiota dysbiosis and increased permeability. Translocation of endotoxin (LPS) via the portal circulation activates hepatic inflammatory pathways (e.g., Kupffer cell/TLR4 signaling), amplifying liver injury and establishing a gut–liver axis of systemic aflatoxicosis.

The intestinal barrier is compromised, and the epithelium is more easily penetrated by bacterial products like LPS [158]. Barrier-compromising experiments in animals have established a mechanism whereby the loss of epithelial integrity promotes bacterial translocation, and liver work has specifically explored this in intestinal permeability and liver bacterial translocation in AFB1-related settings [159]. Minimal noninjury doses of LPS clearly enhanced AFB1 hepatotoxicity in rats, showing that exposure to endotoxins can boost aflatoxin liver toxicity [160]. This biological interaction is important because LPS induces hepatic innate immune response signaling, including, but not limited to, Kupffer cell and TLR4-linked inflammatory signaling, which increases liver damage [161]. In practical terms, AFB1-induced barrier damage can convert a local intestinal insult into a systemic inflammatory syndrome by increasing the host’s exposure to microbial products. This is why intestinal injury, microbiota dysbiosis, and hepatic inflammation often appear together in animal aflatoxicosis rather than as isolated events. Mechanistic overview of AFB1-induced systemic toxicity through gut–liver axis in animals is shown in Figure 2.

### 5.3. Reproductive and Developmental Toxicity

In animal models, AFB1 reproductive toxicity is attributed to the result of oxidative stress, mitochondrial dysfunction, endocrine disruption, and apoptosis in gonadal and embryonic tissues [162]. While the specific phenotype depends on the species, the most common effects of prenatal exposure involve reduction in fertility, decreased gamete quality, loss of ovarian follicles, changes in sex-steroid levels, and fetal growth or structural defects [163]. In males, AFB1 compromises fertility by disrupting spermatogenesis, epididymal maturation, and sperm function [164]. In Swiss albino mice, AFB1 caused a marked drop in fertility, lowered epididymal sperm concentration and motility, increased sperm abnormalities, and increased abnormal cytoplasmic droplet retention, which indicates injury to both sperm production and post-testicular maturation [165]. In male rats, oral AFB1 disturbed the hypothalamic–pituitary–gonadal axis by lowering testosterone and LH and raising FSH and prolactin, and the authors linked these changes to injury in Leydig cells, Sertoli cells, germ cells, and possibly pituitary regulation [166]. Duck reproductive cell studies show that AFB1 damages granulosa cell viability and induces NRF2-linked ferroptotic or oxidative injury, which are highly relevant to ovarian function because granulosa cells support follicle growth, steroidogenesis, and oocyte maturation [167]. In pigs, porcine testicular and Sertoli cell studies also show that AFB1 disrupts testicular development through Ras/PI3K/Akt-related signaling, linking germ cell injury to developmental interference in the male gonad [168]. Bull spermatozoa exposed to AFB1 show impaired proteome profiles, reduced sperm viability, and compromised fertilization competence, indicating that AFB1 can directly reduce male reproductive capacity in cattle [169].

The oxidative stress, endocrine disruption, apoptotic damage, sub-optimal development of follicles, and decreased developmental competence of gametes and embryos are well-documented effects of AFB1 in animal models and are the most prominent observed effects in females [170]. In White Leghorn laying hens, chronic low-dose oral AFB1 had a significant effect on both the small, medium, and large size follicles that were normal and capable of developing into a mature ovule and on the level of follicular atresia and follicular death, indicating a progressive reduction in ovarian reserves and ovulatory potential even when levels of exposure are subclinical for long periods [171]. In breeder hens, reduced egg production, fertility and hatchability were observed from the ingestion of AFB1, revealing a direct effect on reproductive output in poultry, and a decrease in egg quality and AFB1 residues in eggs and tissues were observed, suggesting the possibility of an effect on egg quality and residue transfer [172,173]. Studies on bovine embryo cultures indicated that AFB1 and AFM1 have an impact on embryo development and morphogenetics and that early embryonic progression is sensitive to AFB1 exposure in cattle [174]. Lab studies of ovine oocytes demonstrate a negative effect of AFB1 on nuclear and cytoplasmic maturation and early competence, and the production of ROS and apoptosis indicates that the embryo is at risk from the very beginning at the oocyte stage [175]. Aflatoxin B1 has the potential to affect the production of sex steroids in animals, causing detectable reproductive endocrine disruption. Oral AFB1 decreased LH and increased FSH and prolactin, and estradiol was also decreased at the highest dose, causing a disruption in the pituitary–gonadal signaling in male rats [162,176]. In goats, prolonged oral AFB1 changed the blood pattern of oestradiol-17β and progesterone during both the luteal phase and synchronized oestrus, showing that ruminants also develop measurable endocrine disruption [177,178]. Comparative reproductive and developmental toxicity of AFB1 in animals are shown in Table 4.

### 5.4. Growth Performance and Metabolic Effects

Although growth performance, feed intake, feed efficiency, and nutrient digestibility are frequently evaluated in AFB1 toxicity studies, these parameters should not be considered specific biomarkers of AFB1 exposure. Rather, they represent functional physiological outcomes that reflect the cumulative impact of AFB1 on nutrient utilization, intestinal integrity, hepatic metabolism, and overall animal health. Because these production-related traits are influenced by multiple factors, including genetics, age, nutritional status, management practices, and environmental conditions, their interpretation should be made in conjunction with molecular, biochemical, and histopathological biomarkers obtained under controlled experimental conditions. The studies discussed in this review were selected because AFB1 was the primary experimental variable, thereby minimizing potential confounding factors and allowing these functional outcomes to be interpreted within the context of AFB1-induced toxicity [32,183].

In animal AFB1 toxicology, reduced feed intake and lower weight gain are among the earliest production-level outcomes, and this pattern is already visible in avian and piglet models [184]. That performance loss is consistent with a multiorgan toxic burden in which intestinal injury, hepatic dysfunction, oxidative stress, and inflammation occur together rather than as isolated events [97]. In broilers, dietary AFB1 significantly decreased body-weight gain and feed intake and increased feed conversion ratio, making growth performance one of the most sensitive readouts of avian aflatoxicosis [185,186]. In ducks, AFB1 also reduced body weight and worsened feed conversion efficiency, showing that avian growth suppression reflects a broader hepatointestinal toxic phenotype [187]. In pigs, chronic low-level AFB1 suppresses growth performance and average daily gain and also reduces apparent total tract digestibility [188]. The additional effects of AFB1 on the integrity of the intestinal barrier and on apoptosis contribute to the fact that growth loss can continue even after the supply of feed is available [131]. For juvenile hybrid groupers, dietary AFB1 resulted in dose-dependent growth inhibition and increased feed conversion ratio and liver injury markers, suggesting that decreased growth in biomass is a conserved vertebrate response to aflatoxin stress [189]. The effects of AFB1 on lambs were shown to affect meat quality and to correlate with oxidative stress, inflammation, and gut microbiota disturbance in sheep [190]. In goats, similarly, AFB1 caused losses in total weight gain, average daily gain, and also in nutrient digestibility [191].

AFB1 affects the handling of nutrients in all three pathways (protein, lipid, carbohydrate), and the growth deficit it causes is not just a feeding problem, but also a metabolic reprogramming problem [192]. In dairy goats, low-dose AFB1 affects serum metabolites associated with multiple pathways of central energy and substrate metabolism, a result indicating that these pathways are not only changed, but also involve shifts in more than one nutrient class [193]. In dairy cows, the metabolomic profiling identified metabolites in the rumen fluid, plasma, and milk that were related to AFB1, such as pathways involving amino acids, representing changes in the protein turnover and nitrogen economy at the whole-animal level [194]. Sheep studies show that AFB1 can lower nitrogen retention and reduce the efficiency with which dietary protein is converted into productive tissue, confirming that ruminants also suffer measurable production losses [123]. Taking together, these species differences still point to one common endpoint: AFB1 creates a low-efficiency metabolic state in which feed is consumed, but a smaller fraction is converted into productive output. Comparative growth performance and metabolic effects of AFB1 across animal species are summarized in Table 5.

## 6. Emerging Therapeutic Strategies

**Figure 3:**
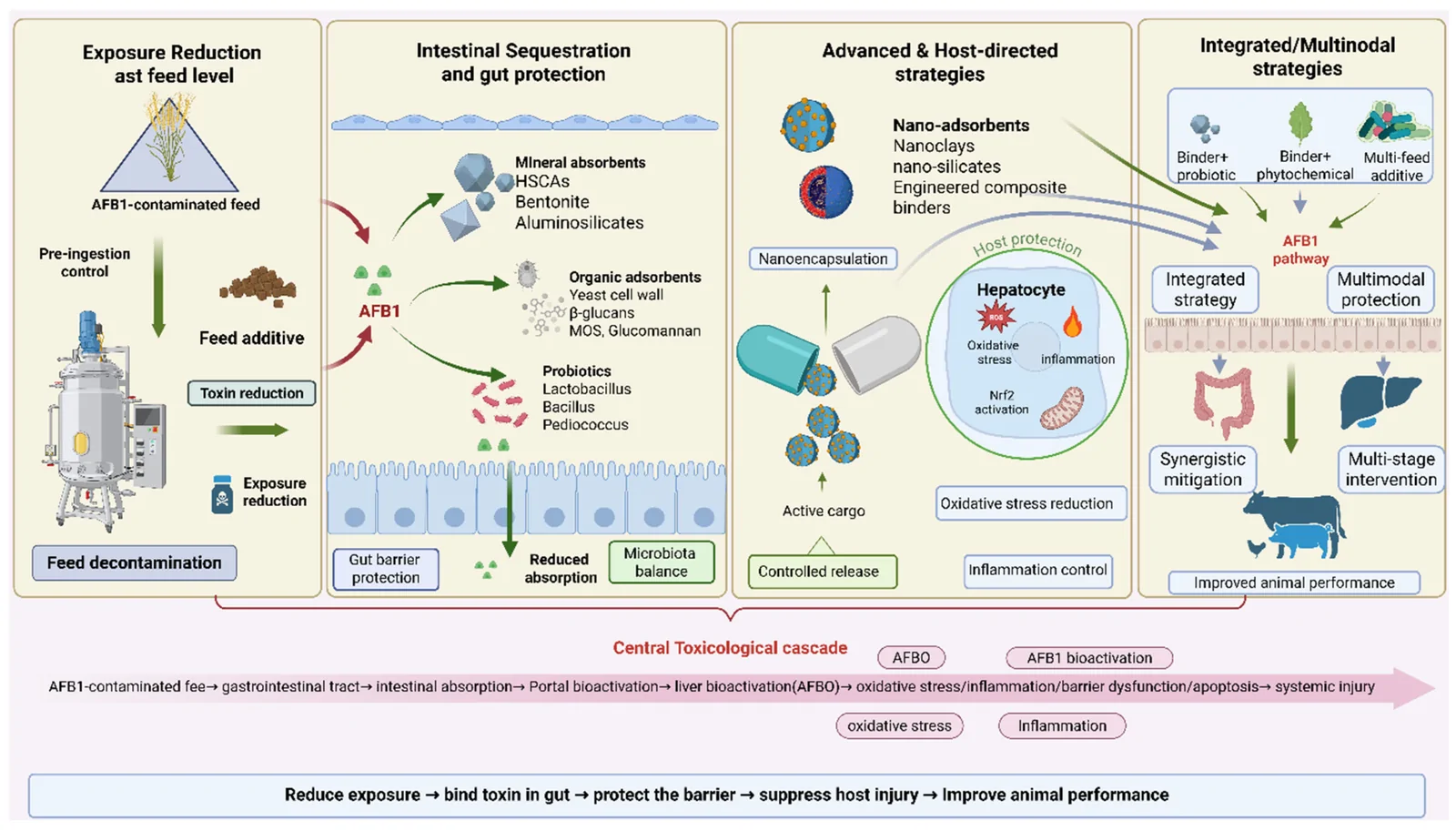
Schematic illustration of a multi-layered intervention framework showing the progression of AFB1 from contaminated feed to systemic toxicity, alongside targeted mitigation strategies at each stage. Pre-ingestion control reduces exposure at the feed level, while intestinal-phase interventions (mineral and organic adsorbents, probiotics) limit toxin bioavailability and preserve gut integrity. Advanced approaches, including nano-adsorbents and nanoencapsulation, enhance binding efficiency and delivery of protective compounds. Host-directed mechanisms attenuate hepatic bioactivation (AFBO formation), oxidative stress, and inflammation. Integrated multimodal strategies provide synergistic protection across multiple stages, ultimately reducing system injury and improving animal health outcomes.

Intervention strategies can target multiple stages of AFB1-induced toxicity, including prevention of gastrointestinal absorption, inhibition of cytochrome P450-mediated bioactivation, enhancement of phase II detoxification, attenuation of oxidative stress and inflammatory signaling, preservation of intestinal barrier integrity, and mitigation of organ-specific injury [22] (Figure 3). This is a stage-based approach that aligns with the animal literature, in which AFB1 toxicity is shown to occur through feed contamination, gastrointestinal absorption, liver activation, oxidative stress, inflammation, barrier injury, and downstream organ dysfunction, rather than as a single isolated lesion [153]. The pre-ingestion stage aims to reduce the available biologically active AFB1 in the feed before the animal consumes it, primarily through feed decontamination, binders, and other additives [196]. Studies indicate that there are interventions on the feed level that can enhance their growth performance and minimize AFB1-related losses, such as clay, yeast, and detoxifiers, all of which serve as barriers to their exposure, the first of which is feed-level mitigation [186]. Given that AFB1 damages intestinal morphology, changes the microbiota, and compromises barrier integrity during the intestinal phase, this process is now seen as an active therapeutic target [60]. In mice, a combination of *Lactobacillus rhamnosus* GG (LGG), *Lactobacillus acidophilus* (LAC), and montmorillonite (MMT) reduced AFB1-induced liver and intestinal injury and improved microbiota disorder, showing that gut-directed interventions can blunt systemic toxicity [105]. A logical intervention strategy is to block the internal activation (hepatic bioactivation) at the level of the cytochrome P450 system that converts AFB1 to the reactive 8,9-epoxide [197]. The metabolism of AFB1 in bovine hepatocyte-like cells showed that CYP1A1 had different metabolic abilities than CYP3A74, with the former linked to the formation of AFM1 and the latter strongly involved in the production of 8,9-exo-epoxide, which leads to cytotoxicity [15]. It is important that the species-specific biotransformation pattern is considered because the same intervention can have different effects in poultry, pigs, ruminants, and rodents [22]. Commercial poultry species, particularly turkeys and ducks, are generally among the most susceptible domestic animal species to AFB1, whereas susceptibility varies considerably among avian species and even among different turkey genotypes owing to differences in hepatic biotransformation and detoxification capacity. The outputs at the system injury stage are oxidative stress, inflammation, apoptosis, and barrier failure, and AFB1-induced dysbiosis may exacerbate liver inflammation at this stage [198].

Feed-level prevention or decontamination measures aim to reduce the amount of biologically available AFB1 before it reaches the animal at the pre-ingestion stage [199]. Among the clays, HSCAS and related aluminosilicates are most extensively studied and continue to be considered practical and field-ready feed-borne aflatoxin binders in production animals [200]. More recent broiler studies also demonstrated that supplementation using HSCAS will enhance growth and health in AFB1-challenged birds and support the use of HSCAS as a viable and practical mitigation tool [201]. Regarding clay chemistry, the AFB1 binding is associated with the interaction with surface functional groups and changes in crystallinity, pore structure, ion substitution, and lattice strain, which are properties that make these materials suitable as sorbents, but not necessarily as hepatic enzyme modulators [202]. In broilers, 0.5% HSCAS reduced the growth depression and liver damage caused by the presence of aflatoxin, and also increased the indices of serum proteins like albumin and total protein [203]. In a separate broiler residue study, 0.5% HSCAS reduced AFB1 and its metabolites in the liver and kidney, but did not totally remove the lesions from liver (and kidney) tissues [204]. Protection was also shown in older turkey studies, with an observed dose-responsive protective effect for all poultry models at approximately 0.5% HSCAS [205]. In dairy cows, certain binders proved to be much more effective in blocking the transfer of AFM1 into milk than others, suggesting that the efficacy of a binder is species- and product-specific and is not a universal trait [206].

Organic adsorbents are considered an important pre-absorption tool as they reduce the exposure of AFB1 in the gut and also promote intestinal health, and thus limit the activation of the systemic toxic cascade [153]. The most exemplified are products from the cell wall of yeast, such as β-glucans, mannans, mannan oligosaccharides, etc., which have been reviewed recently as practical feed additives; they are biodegradable and can interact with adsorption with host support, not acting as an inert binder only [207,208]. At a mechanistic level, binding to yeast cell walls is a two-fold process: binding is mediated by adsorption sites on the surface of polysaccharide-rich yeast cells, but broiler studies have shown that the binding of AFB1 may be mediated by hydrogen bonding, van der Waals forces, and ionic interactions, and that the structure of β-glucan plays an important role in sequestration [209]. In broiler chickens challenged with AFB1, supplementation with a fraction of yeast cell wall (YCWF) enhanced weight gain and feed conversion ratio, increased villus height, villus area, crypt depth, number of goblet cells, and decreased mortality and intestinal damage, demonstrating non-in vitro activity of YCWF in toxin binding [210]. In another broiler experiment, mannan oligosaccharides significantly increased ADFI, ADG, and FCR when challenged with aflatoxin, and decreased the populations of *Escherichia coli*, *Salmonella*, and *Klebsiella* in the ileum and restored some intestinal morphology, indicating the presence of both adsorption and microbiota effects [211]. Organic adsorbents have also been found to be useful in ruminants, as in dairy ewes fed with aflatoxin-contaminated feed; a modified yeast cell wall extract increased the excretion of aflatoxins in the feces after the animals had been fed for an extended period of time, while it decreased the urinary excretion of AFM1, suggesting lower absorption and higher elimination in the gut [212]. The evidence for polysaccharide-based binders like β-mannan hydrolysates is more promising but less consistent, with a recent pig study showing that the net binding of β-mannan hydrolysate was significantly less than for the clay-based additives, which performed best [213].

Probiotics are increasingly considered as functional biotherapeutics in the control of aflatoxins, as they can bind or degrade AFB1, decrease the absorption in the gut, increase the elimination of toxins in feces, and help to stabilize the gut barrier, gut microbiota, and redox balance [214]. Lactobacillus, Pediococcus, and Bacillus are the genera commonly screened for their AFB1 detoxification capabilities in the current probiotic literature and have recently been proposed as biological detoxifiers and not as typical feed additives [215]. The binding step is mostly an adsorption phenomenon at the cell wall level, since the ability of the probiotic to bind AFB1 efficiently is influenced by the cell wall structure, which includes the presence of teichoic acids and other strain-specific components of the cell wall [216]. In the Lactobacillus casei Shirota study, the binding was dose-dependent, with 98% binding being produced by live cells, while the same rat study demonstrated a decrease in circulating AFB1 and a corresponding alleviation of growth suppression caused by AFB1 [151]. In case of Lactobacillus strains, the evidence is strongest for its effect in broilers, which showed significant benefits on growth performance, liver function, meat quality, antibody and IFN-γ response against challenge with Salmonella in the presence of AFB1 [154]. In a more recent study in geese with SNK-6, L. salivarius SNK-6 had beneficial effects on growth and intestinal function, reduced intestinal permeability when exposed to AFB1, and helped to reduce liver inflammation [217]. Under practical exposure conditions, a Bacillus-based direct-fed microbial was shown to interrupt the toxic cascade as it was able to drastically reduce the severe performance, biochemical, immunologic and hepatic damage induced by 2 ppm AFB1 in broiler chickens [218]. Pediococcus is promising, but the available in vivo data for AFB1 is less extensive than for Lactobacillus: a chicken-gut isolate study found that Pediococcus pentosaceus was able to bind 36.24% of the AFB1. In a recent detoxification screening, Pediococcus strains were found to have measurable AFB1-removal activity [219]. When considering AFB1 control, probiotics may be best summarized as a dual mechanism, with Lactobacillus as the most advanced group, Bacillus as the fastest growing group, and Pediococcus as an emerging probiotic still in need of further in vivo validation.

Nano-adsorbents can be envisioned as a new kind of traditional binders, as recent reviews focused on nano-materials for aflatoxin studies show that they are emerging mitigation tools with potential for the future [220]. The primary scientific benefit of their use is that when particle size is reduced and composite architectures are constructed, surface area can be increased and more active adsorption sites are formed alongside interaction with the target matrix, making pre-absorption toxin interception more efficient [221]. The recent reviews on mycotoxin decontamination have included nanoparticle adsorbents as newer control measures, but also recognized that adsorption, reactive-species treatment, and biotransformation were different approaches, and that the scale-up and feasibility of practical use in field application of the conventional binders were limiting factors for immediate adoption of this technology [222]. The low cost and high adsorption capacity for AFB1 make sepiolite-based nanostructures very attractive for use in removing AFB1 in engineered systems, as has already been demonstrated for sepiolite nanofiber composites [223]. An example of a toxin binder that is useful for poultry is the bentonite–curcumin toxin binder that decreased AFB1-induced liver damage, enhanced intestinal morphology, increased the expression of CLDN-1 and JAM-2, and gave the greatest protection when used in combination at 90% bentonite and 10% curcumin [224].

Nanoencapsulation is conceptually different from adsorption, which does not aim at sequestrating AFB1 itself but focuses on delivering protection compounds that counteract AFB1 injury [225]. Nano-curcumin has been evaluated in broilers combined with Saccharomyces cell wall, as part of an aflatoxicosis-mitigation strategy, with positive effect on the aflatoxin residues and production-related outcomes. In a later study in 2025, free curcumin was tested to be more effective than nano-curcumin on some endpoints [226,227]. The hepatotoxicity effects of AFB1 have also been demonstrated in the broiler model, with curcumin shown to reduce expression and activity of CYP450 and increase the expression of DNA methyltransferase enzyme and overall DNA methylation, reaching the internal target nanoencapsulation aims to deliver more effectively [228]. In recent studies on broilers, it was demonstrated that curcumin was able to ameliorate the liver and intestinal damage induced by AFB1 by activating the Nrf2 pathway, and also by decreasing intestinal permeability, suggesting a dual mechanism of action of nano-encapsulated phytochemicals that involves barrier protection as well as antioxidant activity [229]. The application of nanoencapsulation is practical, as it will protect active compounds that have poor water solubility, low gastrointestinal stability, and variable gastrointestinal bioavailability. It will increase the residence time of active compounds and achieve controlled release within the gastrointestinal tracts and liver. Emerging therapeutic strategies for the mitigation of AFB1 toxicity in animal systems are given in Table 6. The strength of evidence supporting mitigation strategies differs considerably. Clay-based adsorbents and yeast-derived products have been extensively evaluated under controlled feeding conditions and are already applied in commercial livestock production. In contrast, many probiotics, phytochemicals, enzyme-based detoxification systems, and nano-enabled interventions remain supported primarily by laboratory or experimental animal studies. Their long-term efficacy, safety, nutrient interactions, economic feasibility, and regulatory acceptance require further evaluation before widespread field implementation. Accordingly, mitigation strategies should be interpreted according to their current level of evidence rather than assuming equivalent practical readiness. The efficacy of mitigation strategies should be interpreted in the context of study quality, experimental conditions, and practical applicability. Although numerous adsorbents, probiotics, phytogenic compounds, and biological detoxification approaches have demonstrated promising efficacy under controlled experimental conditions, their effectiveness may vary under commercial production systems. Furthermore, clay-based adsorbents differ substantially in AFB1-binding capacity according to their mineral composition, physicochemical properties, and gastrointestinal conditions. Some inorganic adsorbents may also interact with essential nutrients, vitamins, or trace minerals, highlighting the importance of evaluating both efficacy and safety before routine application. Regulatory approval and permitted inclusion levels also vary among countries and should be considered when selecting mitigation strategies for livestock production.

## 7. Future Perspective and Conclusions

Moving forward, animal studies of the toxicity of AFB1 should shift from descriptive to mechanism-specific, stage-specific prevention and intervention. One of the key challenges is the development of standardized panels of species-specific biomarkers, which include exposure markers, early injury indices, markers of oxidative stress, markers of inflammation, markers of apoptosis, and pathway-specific markers including Nrf2 and NF-κB. Integrated panels would aid in early diagnosis and enable more comparisons between poultry, pigs, ruminants, rodents, and other models, and would facilitate more reliable assessment of mitigation strategies. The establishment of longitudinal studies that document the progression from subclinical exposure to system injury is also critical: most economic losses in livestock probably occur prior to the clinical disease’s noticeable symptoms. Future comparative studies integrating toxicokinetics, metabolic enzyme profiling, and exposure biomarkers across multiple animal species are needed to improve the translation of experimental findings to livestock health and risk assessment.

The second priority is to increase the number of multimodal therapeutic interventions targeting various stages of the AFB1 cascade. The diversity of adsorbents, probiotics, enzymatic detoxifiers, and systems based on nanotechnology allows each system to target only one aspect of the toxic process, while combined systems could offer broader protection by decreasing bioavailability, preserving the integrity of the gut, limiting hepatic bioactivation, and supporting antioxidant and anti-inflammatory defense. Under realistic feeding conditions, such as mixed mycotoxins, variable mycotoxin loads, and developmental stages, future studies should compare the effectiveness of single and combined interventions.

Last but not least, more attention needs to be given to the process of translating validation. A large number of promising approaches have yielded good results in experimental studies, but have not yet been tested in field conditions, particularly in species with different digestive physiology and metabolic susceptibility. Further research is also needed to determine the safety, sustainability, and feasibility of the use of advanced binders, probiotics, and nano-formulations over the long term. This should be seen as a systems-level challenge, with the issue of AFB1 mitigation being one of several additive challenges that must be coordinated between feed safety, gut health, hepatic protection, and precision animal nutrition.

The absorption, hepatic bioactivation, oxidative stress, inflammation, barrier dysfunction, apoptosis, and systemic metabolic injury pathways are all linked together, making AFB1 one of the most important feed-borne toxins in animal production, responsible for disrupting health. The toxic effect is influenced by different exposures, biotransformation, detoxification, and sensitivity of tissues; therefore, the biomarkers and clinical effects are seen to be varied between poultry, pigs, ruminants, rodents, rabbits, and fish. This review points out that AFB1 toxicity is multisystemic and not just liver-specific because it causes growth retardation, metabolic dysfunction, immune deficiency, reproductive failure, and developmental incompetence.

This review provides an integrated perspective of exposure biomarkers, early injury markers, mechanism-linked pathways, systemic effects, and emerging therapies for aflatoxicosis in animals. The evidence suggests the best future interventions should be stage-specific, species-specific, combinational, and capable of lowering internal exposure, keeping the intestinal and hepatic function intact, and sustaining productive performance. This approach facilitates transition from ‘reactive treatment’ to ‘proactive prevention’ of AFB1 toxicity in animal production systems.

## Figures and Tables

**Table 1:** Animal matrices used for monitoring AFB1 exposure biomarkers and their practical interpretation.

Animal Species/Model	Matrix Analyzed	Biomarker(s) Measured	Main Interpretation in Animal Studies	Practical Interpretation	Ref.
Broiler chickens	Excreta and ileal content	AFB1-N7-Gua, AFM1	AFB1-N7-Gua was detected in excreta but not ileal content, while AFM1 was detected in both matrices, indicating that excreta is a strong matrix for short-term metabolite recovery in poultry.	Best for **recent exposure** and for studying excretion routes in poultry.	[78]
Pigs	Serum, plasma, urine, feces	AFB1-lysine, AFM1, AFB1	Serum and plasma AFB1-lysine are validated exposure markers in swine, and urinary AFB1/AFM1 can quantify absorbed dose after feeding trials.	Best for **internal dose** and **metabolic activation** in monogastric livestock.	[81]
Dairy cattle	Blood, serum, milk	AFB1-albumin adducts, blood AFB1-DNA adducts, AFM1 in milk	Serum albumin adducts and blood DNA adducts are useful for chronic exposure assessment, while milk AFM1 provides an additional downstream indicator of transfer into milk.	Best for **chronic exposure** and **food-chain relevance** in ruminants.	[63]
Rodent models	Urine, liver, serum	AFB1-N7-Gua, albumin adducts, DNA adducts	Rodent studies show that urinary AFB1-N7-Gua reflects hepatic DNA adduct burden and can be used as a noninvasive marker of genotoxic internal dose.	Best for **mechanistic toxicology** and **DNA damage** studies.	[90]
Comparative animal models	Plasma, urine, excreta	Multi-biomarker panels	Multi-biomarker strategies improve interpretation because metabolites reflect recent exposure; DNA adducts reflect genotoxic dose, and albumin adducts reflect longer-term exposure.	Best for **cross-species comparison** and **detoxifier efficacy** studies.	[91]

**Table 2:** Early injury biomarkers of AFB1 toxicity in animal studies.

Biomarker	Main Biological Meaning	Typical Change in Animal Studies	Mechanistic Interpretation	Ref.
ALT	Marker of hepatocellular membrane leakage and early liver injury.	Increases after AFB1 exposure.	Reflects cytosolic enzyme escape from damaged hepatocytes and early loss of membrane integrity.	[109]
AST	Marker of hepatocellular and mitochondrial injury.	Increases after AFB1 exposure.	Suggests deeper cellular injury and mitochondrial involvement beyond simple membrane leakage.	[123]
ALP	Marker of hepatobiliary and canalicular dysfunction.	Increases after AFB1 exposure.	Indicates disturbance of bile-associated function and hepatic excretory handling.	[104]
MDA	End product of lipid peroxidation.	Increases after AFB1 exposure.	Shows that reactive oxygen species have damaged membrane lipids and amplified oxidative stress.	[99]
GSH	Major intracellular thiol antioxidant and detoxification substrate.	Decreases after AFB1 exposure.	Indicates exhaustion of antioxidant reserves and reduced capacity for glutathione-dependent detoxification.	[124]
SOD	First-line enzymatic defense against superoxide radicals.	Decreases after AFB1 exposure.	Shows weakening of the primary enzymatic barrier against reactive oxygen species.	[115]
CAT	An enzyme that removes hydrogen peroxide.	Decreases after AFB1 exposure.	Indicates impaired control of peroxide accumulation and progression of oxidative injury.	[125]

**Table 3:** Mechanism-linked biomarkers of AFB1 toxicity in animal studies.

Biomarker Group	Key Markers	Main Mechanism After AFB1 Exposure	Typical Animal-Study Pattern	Biological Interpretation	Ref.
Inflammatory cytokines	TNF-α, IL-1β, IL-6, IFN-γ, IL-8, IL-18	AFB1 bioactivation and ROS generation activate innate immune signaling, especially TLR/NF-κB-linked transcription and, in some models, inflammasome-related pathways, leading to cytokine production in liver, intestine, spleen, and other tissues.	Animal studies report increased TNF-α, IL-1β, IL-6, IFN-γ, and in poultry, also IL-8, together with inflammatory tissue injury and immune dysregulation.	It indicates early innate immune activation, inflammatory amplification, and breakdown of immune homeostasis.	[65,127]
Apoptosis markers	Bax, Bcl-2, Bax/Bcl-2 ratio, caspase-3, caspase-9, cytochrome c, p53	AFB1-induced oxidative injury and mitochondrial dysfunction shift the balance toward the intrinsic apoptosis pathway, causing Bax upregulation, Bcl-2 suppression, cytochrome c release, and caspase activation.	Animal studies consistently show Bax ↑, caspase-3 ↑, caspase-9 ↑, p53 ↑, Bcl-2 ↓, and mitochondrial apoptotic injury in liver, intestine, thymus, bursa, kidney, and reproductive tissues.	Reflects mitochondria-mediated programmed cell death and progression from oxidative stress to structural tissue injury.	[113,129,132]
Nrf2/NF-κB-related markers	Nrf2, Keap1, HO-1, NQO1, NF-κB p65, TLR4, MyD88, iNOS	AFB1 often suppresses Nrf2-dependent antioxidant defense while promoting NF-κB-mediated inflammatory transcription; this imbalance favors ROS accumulation, cytokine release, and tissue injury.	Animal studies show Nrf2 ↓, HO-1 ↓, NQO1 ↓, and Keap1 ↑ alongside NF-κB activation; protective interventions restore Nrf2 and suppress NF-κB signaling, and Nrf2-deficient animals are more sensitive to AFB1 toxicity.	Represents the core redox-inflammatory switch that links oxidative stress to cytokine production and apoptosis.	[30,136]

**Table 4:** Comparative reproductive and developmental toxicity of AFB1 across animal species.

Species/Model	Representative Study Dose/Design	Main Reproductive or Developmental Outcome	Statistical Value(s) Reported	Interpretation	Ref.
Broiler breeder hens	Dietary aflatoxin 0, 5, 10 μg/g for 4 weeks.	Fertility was not affected, but hatchability and egg production declined.	Hatchability of fertile eggs during week 1: 95.1% in control, 68.9% at 5 μg/g, and 48.5% at 10 μg/g. Egg production decreased significantly during weeks 3 and 4 in the 10 and 5 μg/g groups.	Clear dose-related impairment of reproductive performance and embryonic survival.	[173]
Pig/porcine oocyte	Acute exposure to 10 and 50 μM AFB1 for 44 h.	Oocyte maturation and developmental competence declined.	Oocyte maturation rate decreased significantly at 50 μM; most oocytes were arrested at GVBD or meiosis I. ROS, LC3, ATG3/5/7, and Bak/Bax/Bcl-xl were increased or altered.	AFB1 directly impairs oocyte maturation through oxidative stress, autophagy, epigenetic change, and early apoptosis.	[179]
Goat	Prolonged oral AFB1 at 50 or 100 μg/day/head for ~1.5 months.	Endocrine-reproductive disruption during luteal phase and synchronized oestrus.	Oestradiol-17β showed a significant negative dependence (*p* < 0.05) on dose; progesterone showed a significant positive dependence (*p* < 0.05) on dose. No significant body weight change or oestrus-duration difference.	AFB1 disrupts the steroid profile even in a ruminant species with partial detoxification.	[178]
Rabbit	Oral gestational exposure on days 6–18 at 0.025, 0.05, and 0.1 mg/kg BW.	Embryotoxicity and teratogenicity.	Crown–rump length was significantly reduced at 0.05 and 0.1 mg/kg; fetal weight was significantly reduced at 0.1 mg/kg; resorptions and post-implantation losses increased at 0.1 mg/kg; minimum oral teratogenic dose was 0.1 mg/kg.	Strong prenatal developmental toxicity with a clear teratogenic threshold.	[180]
Rat	Prenatal exposure to 10, 20, or 50 μg AFB1.	Developmental programming and post-natal growth impairment.	Offspring body weight decreased by 16.3% at birth, 31.6% at 3 weeks, and 7.5% at 3 months in the high-dose prenatal group. Male reproductive studies also reported significant decreases in sperm count, motility, viability, and coiling at selected doses.	Strong evidence for prenatal developmental toxicity and later-life reproductive impairment.	[181]
Mouse	Oocyte/sperm studies and developmental models with AFB1 exposure.	Reduced oocyte quality, sperm quality, and developmental competence.	The study reported a significant reduction in sperm concentration, motility, and oocyte developmental capacity.	Consistent reproductive toxicity, though some exact effect sizes were not visible in the abstract.	[165]
Chicken embryo	Embryonic exposure to AFB1.	Embryotoxicity and reduced hatchability.	Reduced hatchability and embryonic survival; in one protective study, AFB1-related embryotoxicity was partly reversed, and embryo survival increased by 62.5% with intervention.	Embryonic development is highly sensitive to AFB1 in birds.	[182]

**Table 5:** Functional effects of AFB1 on growth performance, nutrient utilization, and metabolic outcomes in experimental animal models. (The parameters summarized in this table represent functional toxicological outcomes rather than AFB1-specific biomarkers. Because production performance and nutrient digestibility may be influenced by multiple biological and environmental factors, the reported findings should be interpreted alongside biochemical, molecular, and histopathological evidence obtained under controlled experimental conditions).

Species/Model	Feed Intake	Body Weight Gain/Growth	Feed Conversion/Efficiency	Digestibility/Nitrogen Balance	Metabolic Interpretation	Ref.
ISA chicks	Not reported in the excerpt.	T2 (8 μg/kg) and T3 (10 μg/kg) reduced BW and ADG on days 21 and 42 compared with control. T1 (5 μg/kg) did not show the same growth depression in the excerpt.	T2 and T3 showed higher F/G on days 21 and 42 compared with control; T2 and T3 also had higher F/G than T1 on day 42.	-	AFB1 produced dose-dependent oxidative stress and inflammatory damage, with lower T-AOC, GPX, SOD, IgA, IgG, and higher MDA; cytokine mRNA changes showed immune activation and inflammatory imbalance.	[195]
Ducks/ducklings	Average daily feed intake stayed close between groups: 29.57 ± 2.34 vs. 28.19 ± 1.56 g in week 1 and 60.50 ± 3.64 vs. 57.50 ± 5.77 g in week 2; these differences were not significant.	Initial weight was similar at the start (50.70 ± 2.15 vs. 50.92 ± 2.38 g). AFB1 then lowered body weight and ADG in both weeks: week 1 BW 178.13 ± 13.71 vs. 161.46 ± 8.27 g; ADG 18.20 ± 2.03 vs. 15.79 ± 1.01 g; week 2 BW 412.92 ± 16.08 vs. 367.08 ± 23.31 g; ADG 33.54 ± 0.73 vs. 28.90 ± 3.45 g	Feed conversion became poorer: week 1 FCR 1.63 ± 0.06 vs. 1.79 ± 0.07, and week 2 FCR 1.80 ± 0.12 vs. 1.99 ± 0.08.	-	AFB1 caused abnormal bile metabolism together with liver cell and mitochondrial injury, lower albumin, total protein, cholesterol, T-SOD, GPx, and GSH, and higher ALT, AST, ALP, TBA, and MDA; hepatic CYP7A1 and SCD/SCD5 went up, while BSEP, HMGCS1/HMGCR, FASN, FADS2, and CAT went down.	[97]
Growing pigs (chronic dietary AFB1 exposure; 280 μg/kg diet for 102 days)	↓ ADFI: 2544.08 ± 41.64 → 2332.18 ± 96.26 g/d (*trend*, *p* = 0.07)	↓ ADG: 927.25 ± 15.69 → 847.16 ± 33.52 g/d (*trend*, *p* = 0.06); final BW: 132.80 ± 2.10 → 124.60 ± 3.43 kg (*p* = 0.07)	F/G: 2.75 ± 0.04 vs. 2.75 ± 0.05 (*p* = 0.91, no significant change)	↓ Apparent total tract digestibility of dry matter (−1.6 to −2.1%; *p* = 0.03–0.04) and gross energy (−1.7 to −2.2%; *p* = 0.01–0.03); ether extract digestibility decreased by 4.7% during the 75–105 kg growth phase (*p* = 0.04), whereas crude protein digestibility was unaffected (*p* > 0.05)	Chronic dietary AFB1 impaired nutrient utilization and intestinal absorptive function through oxidative stress and intestinal barrier dysfunction, resulting in reduced growth performance and feed utilization efficiency.	[25]
Saanen goats	Not reported	Total weight gain ↓ from 1883 g in control to 550 g at 500 μg/kg AFB1, and ADG ↓ from 135 to 39.3 g (*p* = 0.029, linear). Final BW was not significantly different.	No direct FCR was reported, but production efficiency clearly worsened with increasing AFB1 dose.	DM digestibility ↓ from 74.0 to 71.3% (*p* = 0.033); EE ↓ from 67.8 to 62.8% (*p* < 0.001); CP ↓ from 75.2 to 70.9% (*p* = 0.048); OM ↓ from 72.2 to 69.0% (*p* = 0.043); NDF ↓ from 60.9 to 55.3% (*p* = 0.013); ADF ↓ from 58.9 to 51.6% (*p* = 0.004). Retained N ↓ from 16.2 to 12.8 g/d (*p* = 0.002); N retention rate ↓ from 56.2 to 44.5% (*p* = 0.002); N bioavailability ↓ from 74.8 to 62.9% (*p* = 0.018).	Plasma metabolites shifted strongly, with 1338 differential metabolites at 50 μg/kg and 1358 at 500 μg/kg; the most affected pathways were choline metabolism, glycerophospholipid metabolism, and amino acid metabolism. Blood indices also moved with dose: RBC ↑ 19.8 → 22.4, HGB ↑ 103 → 113, MCV ↑ 14.6 → 16.6, and MCH ↓ 5.12 → 4.70.	[191]
Sheep/mutton sheep	↓ DMI (significant decrease, *p* < 0.01)	↓ ADG (trend, *p* = 0.08)	No significant change in FCR	↓ DM, CP, EE digestibility; ↓ N intake and retained N; ↑ urinary N excretion	AFB1 reduces nutrient utilization and protein retention, likely via impaired intestinal function and protein metabolism; induces oxidative stress, liver damage (↑ ALT, AST), and immune suppression, shifting nutrients toward maintenance and detoxification rather than growth	[103]

**Table 6:** Emerging therapeutic strategies for the mitigation of AFB1 toxicity in animal systems.

Strategy	Representative Agents	Main Mechanism	Target Stage	Key Take-Home Point	Ref.
Mineral-based adsorbents	HSCAS, bentonite, aluminosilicates	Physicochemical binding of AFB1 in the gastrointestinal tract; reduced bioavailability before absorption	Pre-ingestion/intestinal	Strongest practical first-line strategy; improves growth and liver-related outcomes, but efficiency depends on mineral type, dose, and species	[201,230]
Organic adsorbents	Yeast cell wall, β-glucans, MOS, glucomannan, polysaccharide binders	Adsorption plus intestinal support; yeast/polysaccharide surfaces provide binding sites and help preserve gut function	Pre-absorption/intestinal	Useful when the goal is both toxin binding and gut support; generally more variable than mineral binders	[212,231,232,233]
Probiotics	*Lactobacillus*, *Bacillus*, *Pediococcus*	Cell wall binding, reduced intestinal absorption, microbiota stabilization, and barrier support	Intestinal	Promising because they act on both toxin handling and gut health; effects are strain-specific	[151,157,215,234,235]
Nano-adsorbents	Nanoclays, nano-silicates, chitosan-based nanocomposites, composite nano-binders	Larger surface area, faster binding kinetics, and improved adsorption stability in gut-like conditions	Pre-absorption	High-potential next-generation binders, but field validation and safety remain important	[236,237]
Nanoencapsulation of protective compounds	Nano-curcumin, nanoformulated phytochemicals, β-cyclodextrin-based systems	Improves solubility, stability, controlled release, and tissue delivery of protective compounds	Hepatic/systemic	Strengthens antioxidant and anti-inflammatory protection rather than binding toxin directly	[226,229,238]

## Data Availability

No new data were created or analyzed in this study. Data sharing is not applicable to this article.
